# Association between single euploid embryo transfer and pregnancy outcomes in women of advanced maternal age: a propensity score-matched analysis

**DOI:** 10.3389/fendo.2026.1921930

**Published:** 2026-09-29

**Authors:** Bojin Qin, Xiaolong Lin, Zengyu Yang, Chun Yang, Liyan Wang, Xiaofei Liu, Junjie Zhong, Liuping Lan, Juguang Zhang, Su'e Wei, Lingling Zhu, Xiang Li

**Affiliations:** Reproductive Medicine Center, Yulin Maternal and Child Health Hospital, Yulin, Guangxi, China

**Keywords:** advanced maternal age, euploid embryos, frozen-thawed embryo transfer, PGT-A, pregnancy

## Abstract

**Objective:**

To explore the correlation between preimplantation genetic testing for aneuploidy (PGT-A) and pregnancy, miscarriage, and live birth outcomes among women of advanced maternal age receiving single blastocyst transfer.

**Methods:**

In this single-center retrospective cohort study, this study included patients who underwent frozen-thawed embryo transfer at the Reproductive Medicine Center of Yulin Maternal and Child Health Hospital from January 2024 to January 2026. Based on whether preimplantation genetic testing for aneuploidy was performed, the patients were categorized into the PGT-A group and the N-PGT-A group. Propensity scores were calculated via logistic regression and followed by 1:1 nearest-neighbor matching with a caliper width set to 0.2 multiplied by the logist of the propensity score, random matching order, and matching without replacement. Matching variables included maternal age, infertility type, BMI, endometrial preparation protocol, endometrial thickness, endometrial morphology, and serum progesterone levels. After matching, 144 cycles were included in each group. The primary outcome was clinical pregnancy rate, and secondary outcomes included embryo implantation rate, biochemical pregnancy loss rate, early miscarriage rate, and live birth rate.

**Results:**

Three stepwise-adjusted logistic regression models were constructed based on the matched cohort, with the N-PGT-A group set as the reference. In the unadjusted model 1, PGT-A was significantly correlated with higher implantation, clinical pregnancy, and live birth rates, as well as lower early miscarriage risk (all P≦0.05), while no significant association was observed between biochemical pregnancy loss. After adjusting for baseline matching covariates in Model 2, PGT-A remained significantly correlated with implantation rate(OR = 2.17, 95%CI:1.31–3.59, P = 0.003) and clinical pregnancy rates (OR = 2.11, 95%CI:1.23–3.64, P = 0.007). No statistically significant associations remained for early miscarriage, biochemical pregnancy loss, or live birth rate (all P > 0.05).In the fully adjusted model 3 incorporating AMH, blastocyst stage, and infertility duration, only the inverse association between PGT-A and early miscarriage rate persisted significantly(OR = 0.31, 95% CI 0.1-0.98, P = 0.047); Implantation rate, clinical pregnancy rate, and live birth rate merely presented marginally favorable non-significant trends (all 0.05< P <0.10), and biochemical pregnancy loss rate remained non-signifiscant between groups. Stratified subgroup analyses by maternal age, paternal age and BMI showed all interaction P >0.05 without effect modification. No significant association between PGT-A and early miscarriage was detected across all stratified subgroups.

**Conclusion:**

For female participants aged 35 years or older undergoing single frozen-thawed blastocyst transfer, transfer of PGT-A-screened euploid blastocysts was significantly associated with a decreased risk of early miscarriage. Nevertheless, no statistically significant associations were identified between euploid embryo transfer and clinical pregnancy rate, Biochemical pregnancy loss rate, or live birth rate per single transfer.

## Introduction

Chromosomal aneuploidy represents a major cause of failure in assisted reproductive technology (ART), commonly leading to arrested embryo development, implantation failure, and early pregnancy loss (1). Aneuploidy typically results from chromosome segregation errors during gametogenesis, with trisomy being the most frequently observed abnormality (2). Notably, the majority of embryonic chromosomal anomalies are maternal in origin and exhibit a strong association with advancing maternal age. A study involving 1,501 embryos reported an overall aneuploidy rate of 46.8%, with rates increasing from approximately 28% in women under 30 years to about 67% in those over 40 years of age (3).

Preimplantation genetic testing for aneuploidy (PGT-A) represents a key embryological strategy for selecting chromosomally normal embryos before transfer. While PGT-A has been widely integrated into clinical practice, evidence regarding its impact on reproductive outcomes remains inconsistent. A prospective observational study of 2,538 patients reported higher per-transfer live birth rates with PGT-A (4). Conversely, other studies have indicated that PGT-A may not improve clinical outcomes in the general infertile population but could increase live birth rates specifically in women over 35 years of age (5). Notably, reproductive outcomes following euploid embryo transfer demonstrate significant age-related variation. Meta-analytic data suggest live birth rates per euploid blastocyst transfer generally range from 50% to 60%, with some reports showing no benefit-or even decreased cumulative live birth rates-associated with PGT-A (6). Female participants aged 38 years or older at oocyte retrieval exhibited significantly lower live birth rates following both single and double euploid embryo transfers compared to younger patients (7).** A** large 2020 retrospective study evaluating over 8,000 single euploid embryo transfers further substantiated that advancing female age continues to adversely affect live birth rates even with euploid embryos (8). Given these persistent uncertainties, the study aims to evaluate associations between PGT-A and reproductive outcomes among advanced-age patients with single blastocyst transfer, and provide reference for clinical application of PGT-A.

## Materials and methods

### Study design and participants

This single-center retrospective cohort study was conducted at the Reproductive Medicine Center of Yulin Maternal and Child Health Hospital. Women who underwent frozen-thawed embryo transfer (FET) cycles between January 2024 and January 2026 were screened for inclusion. Eligible patients were stratified into two groups based on the use of preimplantation genetic testing for aneuploidy: a PGT-A group and a N-PGT-A group (**Figure 1**). The inclusion criteria were as follows: single blastocyst FET cycle. Exclusion criteria comprised: (1) Presence of uterine abnormalities (leiomyoma, uterine malformation, adenomyosis, or multiple endometrial polyps); (2) Thin endometrium (endometrial thickness < 7 mm on the day of transfer); (3) blastocysts not derived from 2PN zygotes; (4) Female age < 35 years. The study was approved by the Ethics Committee of Yulin Maternal and Child Health Care Hospital (Ethics Approval No: YLSFYLLKY2025-04-07-01). The study protocol adhered to the ethical principles of the Declaration of Helsinki.

**Figure 1:**
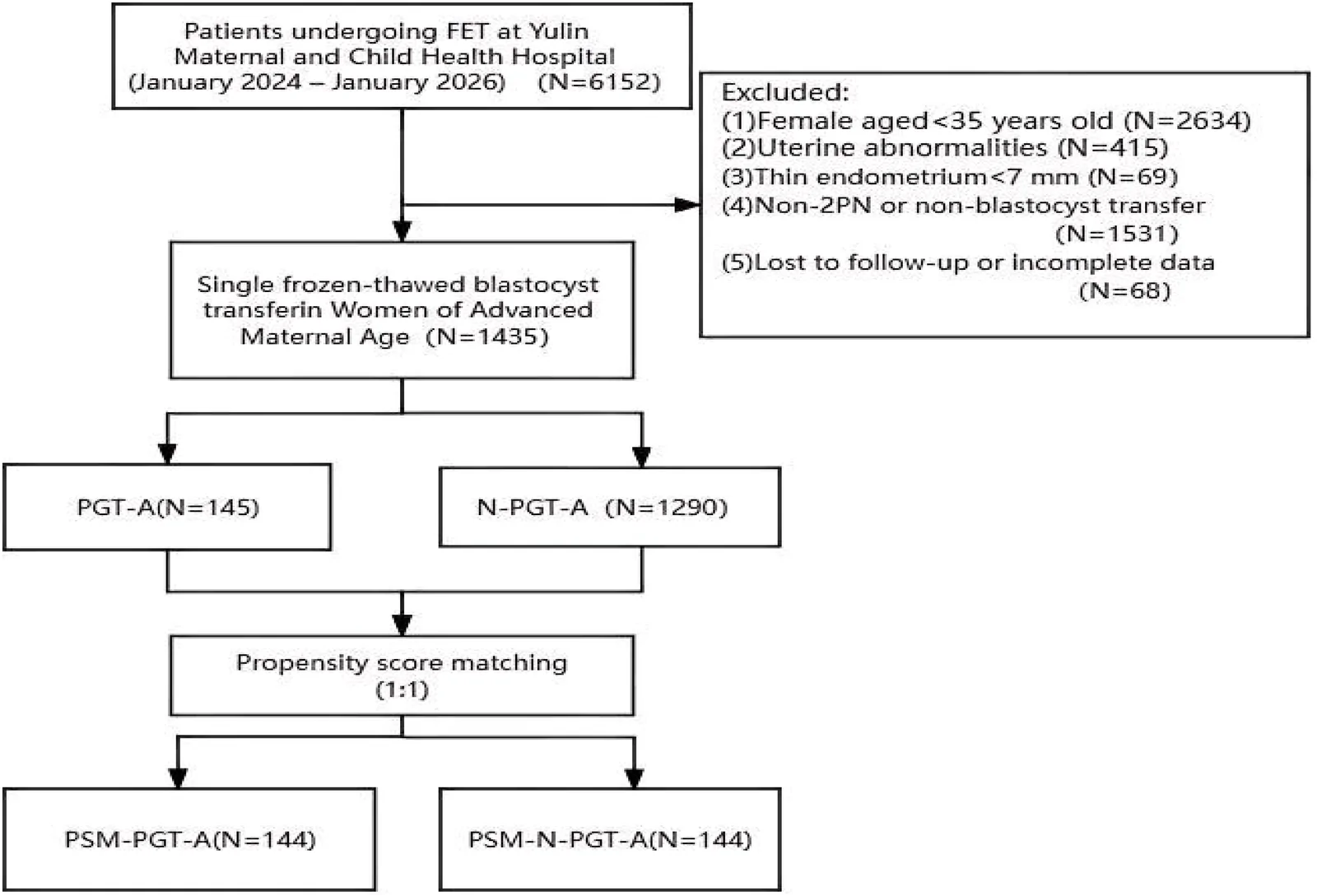
Flow chart of participants.

### Blastocyst grading

All the blastocysts were scored according to the Gardner score standard, according to the blastocyst expansion degree, the development degree of the inner cell mass, and the number of trophoblast cells. Normal fertilization origin, developmental grade ≥ 3, internal cell mass and trophoblast cell grade without C grade, were high-quality blastocysts (3/4/5/6 AA/AB/BA/BB); The internal cell mass and trophoblast cells were classified as C grade, which were usable blastocysts (3/4/5/6 AC/BC/CA/CB).

### Euploidy screening

In cycles undergoing PGT-A, fertilization was achieved via intracytoplasmic sperm injection (ICSI). Normal fertilization was assessed 16–20 hours post-insemination by the presence of two pronuclei (2PN). All embryos derived from 2PN zygotes were cultured to the blastocyst stage (day 5 or 6). Blastocysts were graded according to Gardner score standard. Trophectoderm biopsy was performed preferentially on usable blastocysts with an expansion grade of ≥ 4. In the absence of such blastocysts, biopsy was performed on usable blastocysts with an expansion grade of 3. Following biopsy, all blastocysts were vitrified and stored while awaiting PGT-A results.

### Preimplantation genetic testing for aneuploidy (PGT-A)

All biopsy procedures were performed by experienced embryologists. To avoid mechanical damage to the inner cell mass (ICM), the ICM was positioned at the 8 or 10 o’clock orientation under microscopy. A 10 μm aperture was created on the zona pellucida at the 3 o’clock position using an MTG laser with a pulse duration of 1.0–1.2 ms, followed by aspiration of 5–9 trophectoderm cells. The holding pipette and biopsy pipette were thoroughly washed after the biopsy of each individual embryo to eliminate cross-contamination. After trophectoderm biopsy of eligible blastocysts, whole-genome copy number variation (CNV) amplification was conducted on embryonic cells; samples passing quality control with an amplification success rate above 90% were subjected to library construction and next-generation sequencing (NGS); chromosomal segmental duplications or deletions spanning ≥ 4 Mb were defined as abnormal CNVs, while mosaic variants with affected fragments ≥ 10 Mb and a mosaic proportion ≥ 30% were recorded as mosaic aneuploidy. Embryo ploidy status was finally interpreted and formal PGT-A reports were generated strictly in accordance with these predefined chromosomal variant cut-offs. Only euploid blastocysts were selected for embryo transfer; all fully aneuploid embryos were discarded. Mosaic embryos were cryopreserved or discarded based on patients’ clinical decisions and informed consent. All biopsied blastocysts achieved a 100% survival rate after vitrification and warming, with no statistically significant difference in post-thaw survival compared with non-biopsied blastocysts.

### Endometrial preparation and luteal phase support

Natural Cycle: Transvaginal ultrasound monitoring was initiated on day 10 of the menstrual cycle. When the leading follicle reached ≥ 16 mm, with serum estradiol (E_2_) > 150 pg/mL, progesterone (P) < 1.5 μg/L, and luteinizing hormone (LH) ≥ 20 IU/L, human chorionic gonadotropin (hCG 4000–10,000 IU) or recombinant HCG (250 µg) was administered to trigger ovulation.

Hormone Replacement Therapy Cycle: Oral estradiol valerate 2 mg twice daily was started on menstrual cycle day 2 - 3. After 4 days, the dosage was increased to 4 mg twice daily. Following 12 days of estrogen therapy, transvaginal ultrasound was performed; if endometrial thickness reached ≥ 8 mm, progesterone was administered for secretory transformation.

GnRH-a+HRT Cycle: A long-acting GnRH agonist (1–3 doses) was administered beginning on menstrual cycle day 2–3. Twenty-eight days after the last GnRH-a dose, endometrial preparation followed the HRT protocol described above.

Ovulation Induction Cycle: Letrozole 2.5 mg twice daily was administered for 5 days, starting on menstrual cycle day 2–5. When the leading follicle diameter was ≤ 10 mm, human menopausal gonadotropin (HMG) 75–150 IU was administered every other day. Monitoring and triggering criteria thereafter.

Luteal Phase Support: Following ovulation, endometrial transformation and luteal support were achieved with either oral dydrogesterone and intramuscular progesterone, or oral dydrogesterone and vaginal progesterone. Blastocyst transfer was performed on the fifth day after progesterone initiation.

### Observational indicators and follow-up

Biochemical pregnancy loss was defined as a serum β-hCG level > 5 mIU/mL 12 days after blastocyst transfer, followed by ultrasound confirmation of no intrauterine or extrauterine gestational sac two weeks later. Clinical pregnancy: At least one pregnancy sac (excluding ectopic pregnancy) was detected by B-ultrasound 28 days after transplantation. Early miscarriage rate: pregnancy before 12 weeks of gestation. Live births are defined as newborns who reach 28 weeks of gestation and have vital signs. Clinical pregnancy rate = number of clinical pregnancy cycles÷ number of transplant cycles × 100%. Biochemical pregnancy loss = number of biochemical pregnancy cycles ÷ number of transplant cycles × 100%. Implantation rate = number of gestational sacs ÷ number of embryos transferred × 100% (single blastocyst transfer The number of gestational sacs is only counted as 1). Early miscarriage rate = number of early abortions ÷ number of clinical pregnancies; live birth rate: number of live births ÷ number of transplant cycles × 100%. The latest recorded live birth delivery date was January 17, 2026, and the unified follow-up cut-off time was early February 2026. Overall, 42 transfer cycles failed to achieve sufficient follow-up duration to confirm final live birth outcomes, among which 16 cycles lacked adequate follow-up to evaluate early miscarriage events. Different denominators were applied for the calculation of distinct reproductive endpoints. Specifically, the early miscarriage rate was computed based on clinical pregnancies with complete early pregnancy follow-up, including 81 clinical pregnancies in the PGT-A group and 64 in the N-PGT-A group. The live birth rate was calculated using transfer cycles with full delivery follow-up, with 113 cycles in the PGT-A group and 133 cycles in the N-PGT-A group.

### Statistical analysis

The statistical analysis was performed using Free Statistics software 2.3 based on R language, and the normality of variables was evaluated using the Kolmogorov-Smirnov test. Normally distributed continuous variables are expressed as mean ± standard deviation, while non-normally distributed (skewed) variables are presented as median with interquartile range. Continuous variables were compared using the Student’s t-test or Mann-Whitney U test, as appropriate. Categorical variables, expressed as frequencies and percentages, were analyzed using the chi-square test or Fisher’s exact test. No missing values were observed for outcome variables; the proportion of missing values for covariates was less than 5%, a mild level of missing data. Continuous variables were filled with median values.

Propensity score matching (PSM) was used to balance the groups. Euploid embryo status served as the independent variable, with clinical pregnancy as the outcome variable. The confounding factors included female age, male age, endometrial preparation protocol, infertility type, BMI, endometrial thickness, endometrial morphology, and progestogen levels prior to transplantation. Propensity scores were calculated via logistic regression and followed by 1:1 nearest-neighbor matching with a caliper width set to 0.2 multiplied by the logist of the propensity score, random matching order, and matching without replacement. The matched data were analyzed using univariate analysis and multivariate regression analysis to explore the correlation between various factors and pregnancy outcomes.

Multivariate regression analysis was employed to further examine the relationship between euploid embryos and pregnancy outcomes. Clinically relevant confounding factors and variables showing statistical significance (p < 0.05) in univariate analysis were included in the model. Three analytical models were constructed: Model 1 was the crude unadjusted model. Model 2 was adjusted for female age, male age, BMI, history of recurrent pregnancy loss, endometrial morphology (stratified as A-B, B, B-C, C), and high-quality blastocyst status. Model 3 additionally incorporated AMH, fertilization method, blastocyst stage, and duration of infertility based on model 2. Stratified subgroup analyses were performed by female age, male age, and BMI, and the likelihood ratio test was applied to assess interaction effects across subgroups.

## Results

### Baseline characteristics of the study population

Prior to matching, 145 cycles were included in the PGT group and 1,290 in the N-PGT group. Before matching, significant intergroup discrepancies were observed in male age, female age, endometrial preparation protocol, infertility type, and pre-transfer progesterone. Although SMD values for these covariates decreased markedly after matching, several variables still retained SMDs greater than 0.10. To mitigate bias introduced by this residual imbalance, all such imbalanced covariates were additionally adjusted for in the subsequent conditional logistic regression models (**Table 1**).

**Table 1:** Baseline characteristics of the study population.

Variable	Before				After			
	NPGT-A (n=1290)	PGT-A (n=145)	SMD	p	PSM-N-PGT-A (n=144)	PSM-PGT-A (n=144)	SMD	p
Female age (years)	38.0 ± 2.5	38.8 ± 2.4	0.32	< 0.001	38.5 ± 2.5	38.8 ± 2.4	0.102	0.387
Male age (years)	38.9 ± 4.1	40.0 ± 4.5	0.255	0.003	40.0 ± 3.9	39.9 ± 4.4	0.01	0.933
Endometrial preparation protocol, n (%)			0.946	< 0.001			0.043	0.982
Ovulation induction cycle	257 (19.9)	3 (2.1)			3 (2.1)	3 (2.1)		
GnRH-a+HRT	254 (19.7)	64 (44.1)			60 (41.7)	63 (43.8)		
HRT	522 (40.5)	73 (50.3)			76 (52.8)	73 (50.7)		
Natural cycle	257 (19.9)	5 (3.4)			5 (3.5)	5 (3.5)		
BMI (kg/m^2^)	23.1 ± 3.1	22.9 ± 2.9	0.062	0.493	22.5 ± 2.8	22.9 ± 2.9	0.124	0.293
Type of infertility, n (%)			0.264	0.008			0.029	0.803
Secondary infertility	1108 (85.9)	136 (93.8)			136 (94.4)	135 (93.8)		
Primary infertility	182 (14.1)	9 (6.2)			8 (5.6)	9 (6.2)		
Endometrial thickness (mm)	10.1 ± 1.8	10.0 ± 1.6	0.611	0.435	9.7 ± 1.6	10.0 ± 1.6	0.081	0.172
Endometrial morphology, n (%)			0.32	0.466			0.102	0.98
A	612 (47.4)	66 (45.5)			68 (47.2)	66 (45.8)		
A-B	309 (24)	41 (28.3)			41 (28.5)	40 (27.8)		
B	280 (21.7)	31 (21.4)			27 (18.8)	31 (21.5)		
B-C	38 (2.9)	5 (3.4)			5 (3.5)	5 (3.5)		
C	51 (4)	2 (1.4)			3 (2.1)	2 (1.4)		
P (ng/ml)	0.2 (0.1, 1.0)	0.1 (0.1, 0.2)	0.255	< 0.001	0.1 (0.1, 0.2)	0.1 (0.1, 0.2)	0.01	0.913

*GnRH-a + HRT, Hormone replacement therapy following GnRH agonist down-regulation; HRT, Hormone replacement therapy; BMI, Body mass index; P, Serum progesterone level on the day of transfer.

### Univariate analysis of frozen-thawed embryo transfer outcomes

Univariate regression was conducted to evaluate factors associated with clinical outcomes following frozen-thawed embryo transfer. Female age, infertility type, endometrial thickness, endometrial morphology, high-quality blastocyst status, fertilization method, recurrent miscarriage, and euploid embryo status, were significantly correlated with pregnancy outcomes (all < 0.05) (**Table 2**).

**Table 2:** Univariate analysis of frozen-thawed embryo transfer outcomes.

Variable	Embryo implantation rate		Biochemical pregnancy loss rate		Clinical pregnancy rate		Early miscarriage rate		Live birth rate	
	OR (95% CI)	P	OR (95% CI)	P	OR (95% CI)	P	OR (95% CI)	P	OR (95% CI)	P
Female age (years)	0.98 (0.89~1.08)	0.662	1 (0.85~1.17)	0.988	0.98 (0.89~1.08)	0.719	1.26 (1.08~1.47)	0.003	0.85 (0.75~0.96)	0.009
Male age (years)	0.97 (0.91~1.02)	0.246	1.01 (0.92~1.11)	0.896	0.97 (0.92~1.02)	0.256	1.09 (0.99~1.19)	0.069	0.9 (0.83~0.98)	0.01
AMH (ng/ml)	1.01 (0.91~1.12)	0.854	1.05 (0.9~1.24)	0.512	0.99 (0.89~1.09)	0.834	0.89 (0.73~1.07)	0.212	1.02 (0.91~1.15)	0.749
Fertilization method	0.52 (0.32~0.86)	0.01	1.27 (0.57~2.8)	0.561	0.51 (0.32~0.81)	0.005	1.33 (0.63~2.81)	0.459	0.49 (0.28~0.88)	0.016
Duration of infertility (years)	0.98 (0.91~1.05)	0.513	0.93 (0.81~1.06)	0.254	1 (0.94~1.07)	0.964	0.99 (0.9~1.1)	0.922	1.03 (0.96~1.12)	0.416
GnRH-a+HRT	0 (0~Inf)	0.98	0.49 (0.05~4.59)	0.533	0.22 (0.02~1.91)	0.169	0.98 (0.1~9.53)	0.987	0.4 (0.05~3)	0.374
HRT	0 (0~Inf)	0.98	0.52 (0.06~4.76)	0.561	0.25 (0.03~2.21)	0.213	1.25 (0.13~11.84)	0.846	0.39 (0.05~2.84)	0.349
Natural cycle	0 (0~Inf)	0.98	0.56 (0.03~10.93)	0.699	0.3 (0.02~3.63)	0.344	2 (0.13~31.98)	0.624	0.14 (0.01~2.52)	0.184
BMI (kg/m^2^)	1.03 (0.94~1.12)	0.548	1 (0.87~1.15)	0.993	1.03 (0.95~1.11)	0.528	1.14 (1.01~1.29)	0.039	0.96 (0.87~1.05)	0.365
Type of infertility	0.97 (0.35~2.72)	0.959	1.31 (0.28~6.07)	0.728	0.92 (0.35~2.46)	0.87	0 (0~Inf)	0.99	2.11 (0.75~5.9)	0.156
Recurrent miscarriage	1.65 (0.9~3.02)	0.104	0.11 (0.01~0.84)	0.033	2.4 (1.34~4.31)	0.003	0.3 (0.11~0.82)	0.019	2.55 (1.35~4.84)	0.004
Endometrial thickness	0.01 (-0.02,0.05)	0.546	0.98 (0.77~1.25)	0.867	1.06 (0.92~1.23)	0.404	0.98 (0.77~1.25)	0.867	0.85 (0.75~0.98)	0.02
Endometrial morphology										
A-B	0.78 (0.43~1.4)	0.402	0.88 (0.34~2.31)	0.796	0.79 (0.45~1.39)	0.416	1.24 (0.51~3.04)	0.637	0.96 (0.51~1.81)	0.899
B	0.54 (0.29~1.03)	0.06	1.28 (0.48~3.39)	0.623	0.45 (0.24~0.84)	0.012	1.65 (0.59~4.62)	0.344	0.45 (0.2~1.02)	0.056
B-C	0.62 (0.16~2.3)	0.471	0 (0~Inf)	0.99	0.95 (0.26~3.53)	0.94	4.47 (0.82~24.34)	0.084	0.6 (0.12~3.02)	0.532
C	0.1 (0.01~0.95)	0.045	0 (0~Inf)	0.993	0.16 (0.02~1.46)	0.104	9461203.9 (0~Inf)	0.985	0 (0~Inf)	0.988
High-quality embryo	1.74 (0.97~3.1)	0.062	0.73 (0.29~1.81)	0.495	1.8 (1.01~3.19)	0.045	0.59 (0.23~1.49)	0.265	1.38 (0.69~2.77)	0.361
Embryo stage	0.97 (0.6~1.58)	0.904	2.12 (0.89~5.01)	0.088	0.76 (0.48~1.22)	0.256	0.8 (0.38~1.68)	0.556	0.94 (0.54~1.64)	0.832
Euploid embryo	2.11 (1.28~3.46)	0.003	0.56 (0.25~1.26)	0.161	2.49 (1.55~4.02)	<0.001	0.39 (0.18~0.83)	0.014	2.32 (1.31~4.1)	0.004

*All categorical covariates used the “N-group” group as the reference group in regression models.

### Multivariate logistic regression analysis of pregnancy outcome between euploid embryo and frozen-thawed embryo transfer

Three sequentially adjusted logistic regression models were established to assess the associations between PGT-A and reproductive outcomes within the 1:1 propensity-score-matched cohort. All multivariable logistic regression models took the N-PGT-A group as the reference group. The PGT-A group (transfer of confirmed euploid blastocysts) was defined as the exposure group to estimate the adjusted odds ratios for reproductive outcomes. In the crude model 1, PGT-A was significantly associated with elevated embryo implantation rate (OR = 2.11, 95% CI 1.28–3.46, P = 0.003), clinical pregnancy rate (OR = 2.43, 95% CI 1.51–3.92, P <0.001), and live birth rate (OR = 2.32, 95% CI 1.31–4.1, P = 0.004), as well as reduced risk of early miscarriage rate (OR = 0.47, 95% CI 0.22-1, P = 0.05). No significant correlation was detected between PGT-A and biochemical pregnancy loss rte (OR = 0.56, 95% CI 0.25-1.26, P = 0.161). After adjustment for basic confounders in Model 2, the significant associations persisted for embryo implantation rate (OR = 2.17, 95% CI 1.31-3.59, P = 0.003), clinical pregnancy rate (OR = 2.11, 95% CI 1.23-3.64, P = 0.007). Live birth rate (OR=1.85, 95% CI 0.96-3.59, P = 0.068), biochemical pregnancy loss showed no intergroup difference (OR = 0.86, 95% CI 0.37-2.03, P = 0.735). In the fully adjusted model 3, a stable significant association was observed between PGT-A screening and reduced risk of early miscarriage after multivariate adjustment. (OR = 0.31, 95% CI 0.1-0.98, P = 0.047). By contrast, only non-significant borderline favorable trends were observed for embryo implantation rate (OR = 1.73, 95% CI 0.94-3.17, P = 0.076), clinical pregnancy rate (OR = 1.81, 95% CI 0.95-3.44, P = 0.072), and live birth rate (OR = 1.63, 95% CI 0.74-3.6, P = 0.226). No significant intergroup association was observed for the rate of biochemical pregnancy loss across all regression models.(OR = 0.74, 95% CI 0.27-2.05, P = 0.559) (**Table 3**).

**Table 3:** Multivariate logistic regression analysis of pregnancy outcome between euploid embryo and frozen-thawed embryo transfer.

Variable		N.event (%)	Model 1		Model 2		Model 3	
			OR (95% CI)	P	OR (95% CI)	P	OR 95% CI)	P
Embryo implantation rate	N-PGT-A	82 (56.9)	1 (Ref)		1 (Ref)		1 (Ref)	
	PGT-A	106 (73.6)	2.11 (1.28~3.46)	0.003	2.17 (1.31~3.59)	0.003	1.73 (0.94~3.17)	0.076
Biochemical pregnancy loss rate	N-PGT-A	17 (11.8)	1 (Ref)		1 (Ref)		1 (Ref)	
	PGT-A	10 (6.9)	0.56 (0.25~1.26)	0.161	0.86 (0.37~2.03)	0.735	0.74 (0.27~2.05)	0.559
Clinical pregnancy rate	N-PGT-A	65 (45.1)	1 (Ref)		1 (Ref)		1 (Ref)	
	PGT-A	96 (66.7)	2.43 (1.51~3.92)	<0.001	2.11 (1.23~3.64)	0.007	1.81 (0.95~3.44)	0.072
Early miscarriage rate	N-PGT-A	21 (32.8)	1 (Ref)		1 (Ref)		1 (Ref)	
	PGT-A	15 (18.5)	0.47 (0.22~1)	0.05	0.48 (0.19~1.23)	0.126	0.31 (0.1~0.98)	0.047
Live birth rate	N-PGT-A	27 (20.3)	1 (Ref)		1 (Ref)		1 (Ref)	
	PGT-A	42 (37.2)	2.32 (1.31~4.1)	0.004	1.85 (0.96~3.59)	0.068	1.63 (0.74~3.6)	0.226

*Model 1: crud Model; Model 2: Female age, male age, BMI, recurrent miscarriage, endometrial morphology (A–B, B, B–C, C), high-quality blastocyst; Model 3: Model 2 + AMH, fertilization method, blastocyst stage, duration of infertility.

*Early miscarriage rates were calculated from clinical pregnancies with complete follow-up for early pregnancy outcomes. (PGT-A: n=81; non-PGT-A: n=64).

*Live birth rate was calculated based on transfer cycles with complete delivery follow-up (PGT-A: n=113; non-PGT-A: n=133).

*N.event (%): Number of positive events in the PGT-A group.

### Subgroup analysis

Subgroup analyses were performed to assess whether female age, male age, and body mass index (BMI) modified the associations of PGT-A with clinical pregnancy rate and early miscarriage risk. No statistically significant interactions were identified for either outcome across all stratifying variables (all P for interaction > 0.05). For clinical pregnancy rate, PGT-A was significantly associated with improved outcomes only in the following subgroups: women aged 41–47 years (OR = 3.24, 95% CI: 1.07–9.81, P = 0.038), male partners aged ≥40 years (OR = 3.12, 95% CI: 1.31–7.45, P = 0.01), and patients with normal BMI (18.5–24 kg/m², OR = 2.34, 95% CI: 1.16–4.74, P = 0.018). No significant intergroup differences were observed in the remaining age or BMI strata. For early miscarriage risk, no statistically significant between-group differences were detected in any subgroup. However, a trend toward reduced early miscarriage risk was noted in the PGT-A group among women aged 41–47 years (P = 0.069), male partners aged ≥40 years (P = 0.097), and patients with overweight BMI (24–28 kg/m²,P = 0.066) (**Table 4**).

**Table 4:** Subgroup analysis.

Variable	Clinical pregnancy rate					Early miscarriage rate			
		N	OR (95% CI)	p	P for interaction	N	OR (95% CI)	p	P for interaction
Female age (years)		288			0.639	145			0.858
35-37	N-PGT-A	58	1 (Ref)			15	1 (Ref)		
	PGT-A	42	1.87 (0.72~4.85)	0.196		17	0.53 (0.08~3.72)	0.526	
38–40	N-PGT-A	52	1 (Ref)			29	1 (Ref)		
	PGT-A	66	1.92 (0.79~4.63)	0.148		36	0.51 (0.14~1.81)	0.295	
41-47	N-PGT-A	34	1 (Ref)			20	1 (Ref)		
	PGT-A	36	3.24 (1.07~9.81)	0.038		28	0.33 (0.1~1.09)	0.069	
Male age (years)		288			0.673	145			0.916
<35	N-PGT-A	8	1 (Ref)			5	1 (Ref)		
	PGT-A	10	1.19 (0.06~22.53)	0.907		6	0.8 (0.04~17.2)	0.887	
35–39	N-PGT-A	67	1 (Ref)			30	1 (Ref)		
	PGT-A	70	1.81 (0.81-4.03)	0.145		39	0.48 (0.14~1.71)	0.259	
≥40	N-PGT-A	69	1 (Ref)			29	1 (Ref)		
	PGT-A	64	3.12 (1.31~7.45)	0.01		36	0.41 (0.14~1.17)	0.097	
BMI (kg/m2)		288			0.888	145			0.72
<18.5	N-PGT-A	11	1 (Ref)			6	1 (Ref)		
	PGT-A	6	3 (0.12~73.65)	0.501		4	0 (0~Inf)	0.997	
18.5-24	N-PGT-A	83	1 (Ref)			35	1 (Ref)		
	PGT-A	87	2.34 (1.16~4.74)	0.018		47	0.59 (0.21~1.66)	0.32	
24-28	N-PGT-A	45	1 (Ref)			20	1 (Ref)		
	PGT-A	45	2.3 (0.84~6.25)	0.103		26	0.29 (0.08~1.08)	0.066	
≥28	N-PGT-A	5	1 (Ref)			3	1 (Ref)		
	PGT-A	6	1 (0~Inf)	0.99		4	0.67 (0.02~18.06)	0.81	

## Discussion

In this retrospective cohort analysis focusing on single frozen-thawed euploid blastocyst transfer, euploid embryo status was significantly associated with a reduced risk of early miscarriage. No statistically significant associations were observed between euploidy and embryo implantation rate, clinical pregnancy rate, or live birth rate per single transfer. Interaction tests revealed no significant interactions between PGT-A and female age, male age, or body mass index (BMI).

At least half of all early miscarriages are attributed to embryonic chromosomal abnormalities (9). Screening euploid blastocysts via PGT-A can improve the success rate of single embryo transfer and shorten the time interval to achieve a live birth (10). Accordingly, within a single oocyte retrieval cycle, ongoing pregnancy rates and live birth rates per transfer may be higher with PGT-A-screened embryos relative to untested embryos (11, 12). By avoiding the transfer of aneuploid blastocysts, PGT-A reduces miscarriage rates (MRs) per clinical pregnancy and elevates live birth rates (LBRs) per embryo transfer (13). Nevertheless, our study revealed that transfer of euploid blastocysts was associated with the early miscarriage rate, whereas no significant association was detected with the live birth rate. Consistent observations have been reported in previous research: significant clinical correlates of PGT-A were identified in analyses stratified per embryo transfer; yet, such associations disappeared when analyses were performed per patient (10). All participants enrolled in this study were women aged over 35 years, and stratified analyses revealed no significant associations between PGT-A and clinical pregnancy rate or early miscarriage rate. A large U.S. study covering 56,469 oocyte retrieval cycles demonstrated that PGT-A was correlated with an increased live birth rate for patients older than 35 years (14). Multiple meta-analyses adopted cumulative live birth rate per oocyte retrieval as the primary endpoint, incorporating data from repeated transfers of surplus cryopreserved euploid embryos, and detected no significant association between PGT-A and live birth rates (6, 15, 16) Live birth represents a composite oumaternal, intmaternal, mined by embryonic, maternal and intrauterine factors. Although PGT-A can prevent pregnancy loss, resulting from chromosomal aneuploidy, maternal conditions related to advanced age-including diminished ovarian reserve, impaired endometrial receptivity and endocrine disorders-still restrict the progression of pregnancy to term delivery.

This study is a single-center retrospective cohort analysis. We excluded 42 cycles with insufficient follow-up to confirm live birth outcomes, including 16 cycles lacking adequate data for early miscarriage evaluation. This exclusion may introduce selection bias, as patients with later transfer timing were systematically omitted, and their baseline fertility characteristics may differ from fully followed cases. Unfinished ongoing pregnancies were not counted toward live birth endpoints, which may underestimate the true live birth rate per single euploid blastocyst transfer. In addition, although propensity score matching was applied to balance baseline confounders, several potential confounding variables were not fully collected, and residual confounding could not be completely eliminated. Moreover, we adjusted for paternal age but lacked consistent records of critical male fertility indicators such as sperm DNA fragmentation and semen parameters, leading to incomplete control of male-related confounders. We only assessed live birth after a single blastocyst transfer without analyzing cumulative live birth rates, so long-term reproductive outcomes remain unclear. Cost data for PGT-A were unavailable, precluding cost-effectiveness evaluation. Multicenter prospective cohort studies are therefore required in the future to comprehensively collect indicators related to uterine status, maternal endocrine function and male fertility, with long-term follow-up of cumulative live birth rates, so as to accurately evaluate the genuine clinical benefits of PGT-A for women of advanced maternal age.

## Conclusion

For female participants aged 35 years or older undergoing single frozen-thawed blastocyst transfer, transfer of PGT-A-screened euploid blastocysts was significantly associated with a decreased risk of early miscarriage. Nevertheless, no statistically significant associations were identified between euploid embryo transfer and clinical pregnancy rate, Biochemical pregnancy loss rate, or live birth rate per single transfer.

## Data Availability

The original contributions presented in the study are included in the article/supplementary material. Further inquiries can be directed to the corresponding author.
